# A case of multiple glucagonomas with no clinical manifestations of excess glucagon despite hyperglucagonemia

**DOI:** 10.1002/deo2.230

**Published:** 2023-03-27

**Authors:** Shogo Amano, Shigeyuki Suenaga, Kaori Hamamoto, Shoko Yada, Takanori Tsuyama, Shuhei Shinoda, Yuya Tanaka, Yoshihiro Takemoto, Eijiro Harada, Katsuya Tanabe, Shunichiro Asahara, Kazunobu Hoshii, Taro Takami

**Affiliations:** ^1^ Department of Gastroenterology and Hepatology Yamaguchi University Graduate School of Medicine Yamaguchi Japan; ^2^ Department of Surgery and Clinical Science Yamaguchi University Graduate School of Medicine Yamaguchi Japan; ^3^ Department of Endocrinology Metabolism Hematological Science, and Therapeutics Yamaguchi University Graduate School of Medicine Yamaguchi Japan; ^4^ Department of Internal Medicine Division of Diabetes and Endocrinology Kobe University Graduate School of Medicine Kobe Japan; ^5^ Department of Molecular Pathology Yamaguchi University Graduate School of Medicine Yamaguchi Japan

**Keywords:** glucagonoma, pancreatic tumor, neuroendocrine tumor, glucagon receptors, pancreatectomy

## Abstract

Herein we report the case of a patient with multiple glucagonomas that have been precisely described with endoscopic ultrasound. A 36‐year‐old woman was referred to our hospital for computed tomography investigation of multiple pancreatic masses. Physical examination was unremarkable; on contrast‐enhanced computed tomography, mass lesions were evident in the head, body, and tail of the pancreas. The mass in the pancreatic head was poorly demarcated and exhibited a faint contrast effect, the one in the pancreatic body was a cystic lesion, and the one in the pancreatic tail was hypervascular. Blood investigations showed that serum glucagon was abnormally high at 7670 pg/ml; glucose tolerance was not impaired. There was no family history that suggested multiple endocrine neoplasia type 1 or von Hippel‐Lindau disease. Endoscopic ultrasound revealed that there were additional masses, which were scattered isoechoic to hyperechoic lesions a few millimeters in size. Ultrasound‐guided fine needle biopsy of the lesion in the pancreatic tail resulted in a diagnosis of a neuroendocrine tumor. Based on these pathologic findings, we performed a total pancreatectomy. A large number of nodules with tumor cells were evident in all cut surfaces of the surgical specimen. Immunostaining was positive for chromogranin A and glucagon, and glucagonoma was therefore diagnosed. It is conceivable that attenuated glucagon action could have contributed to the development of the multiple glucagonomas.

## INTRODUCTION

Pancreatic neuroendocrine tumors are a comparatively rare form of pancreatic tumors, accounting for less than 3% of all pancreatic tumors.^1^ However, advances in diagnostic imaging have increasingly aided the identification of tumors in their early stages. Glucagonoma, a tumor of the pancreatic alpha cells with over‐production of glucagon, is an extremely rare disease, accounting for approximately 1%–2%^2^ of pancreatic neuroendocrine tumors. Due to the high serum glucagon level, most cases of glucagonoma exhibit distinctive symptoms, including weight loss and necrolytic migratory erythema.^3^ However, there are extremely rare asymptomatic cases of hyperglucagonemia due to glucagon receptor gene mutation.^4^ We herein report the case of a young woman with multiple glucagonomas, who exhibited no clinical features associated with excess glucagon action, despite having severe hyperglucagonemia.

## CASE REPORT

**FIGURE 1:**
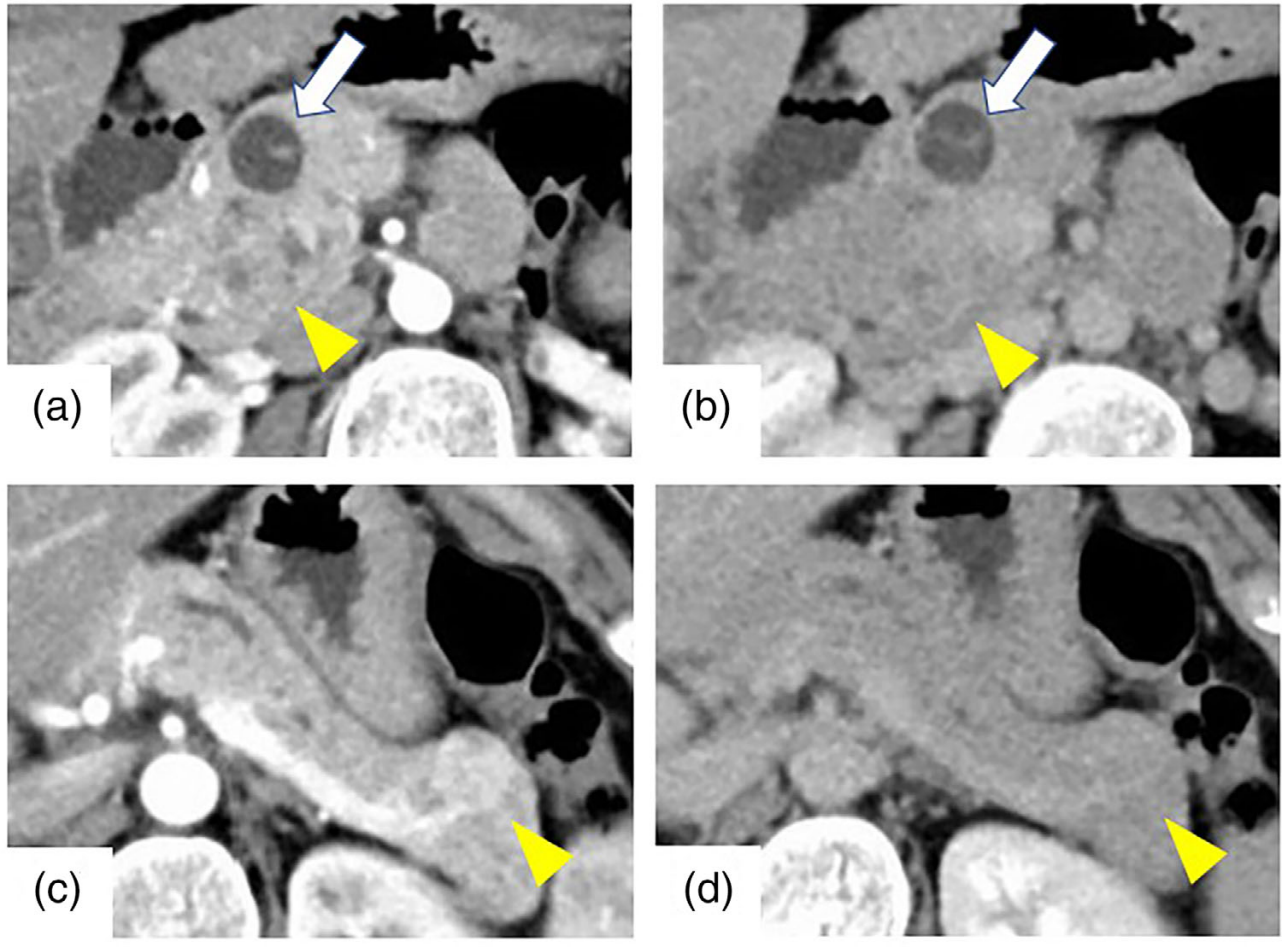
Contrast‐enhanced computed tomography scan. (a) Arterial phase, (b) equilibrium phase: There was a 26‐mm, poorly demarcated, hypodense nodule that exhibited a faint contrast effect in the pancreatic head (yellow arrowhead). There was also a 15‐mm cystic lesion in the pancreatic body (arrow). (c) Arterial phase, (d) equilibrium phase: There was a 17‐mm, well‐demarcated, solid nodule that exhibited a contrast effect during the vascular phase in the pancreatic tail (yellow arrowhead).

**FIGURE 2:**
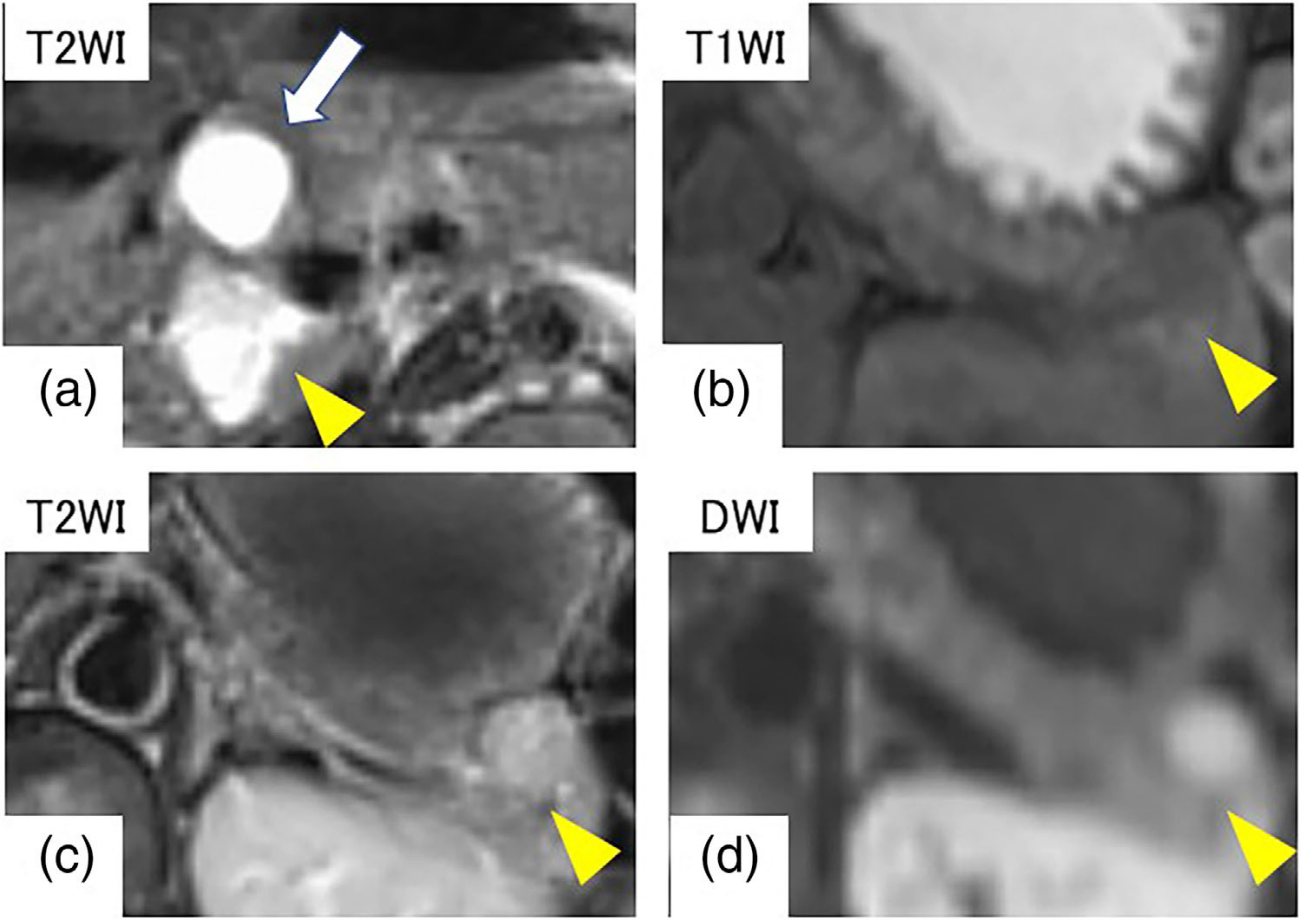
Magnetic resonance imaging. (a) The lesions in the pancreatic head (yellow arrowhead) and body (arrow) were very hyperintense in T2‐weighted imaging. (b–d) The lesion in the pancreatic tail was hypointense in T1‐weighted imaging, hyperintense in T2‐weighted imaging, and hyperintense in diffusion‐weighted imaging.

A 36‐year‐old woman underwent a plain computed tomography (CT) scan to investigate pain in the left breast, which identified a pancreatic mass, and thus, she was referred to our hospital. The patient's medical history was unremarkable, and there was no family history of endocrine disease. On physical examination, her height was 160 cm and weight was 54.3 kg, vital signs were normal, and no abdominal symptoms or cutaneous lesions were present. The patient's blood count and biochemistry test results were within normal limits, and there were no elevated tumor markers. Endocrine hormone tests showed that serum glucagon was abnormally high at 7670 pg/ml. Furthermore, intact parathyroid hormone was only mildly elevated, and glucose tolerance was not impaired. Abdominal dynamic CT (Figure 1) revealed a 26‐mm poorly demarcated, hypodense nodule that exhibited a faint contrast effect in the pancreatic head. There was also a 15‐mm cystic lesion in the pancreatic body, and a 17‐mm clearly demarcated, solid nodule, exhibiting a contrast effect during the vascular phase, in the pancreatic tail. The lesions in the pancreatic head and body were extremely hyperintense on T2‐weighted magnetic resonance imaging (MRI; Figure 2). The solid nodule in the pancreatic tail was hypointense on T1‐weighted imaging and hyperintense on T2‐weighted and diffusion‐weighted imaging. Cranial MRI did not reveal any obvious pituitary lesions.

**FIGURE 3:**
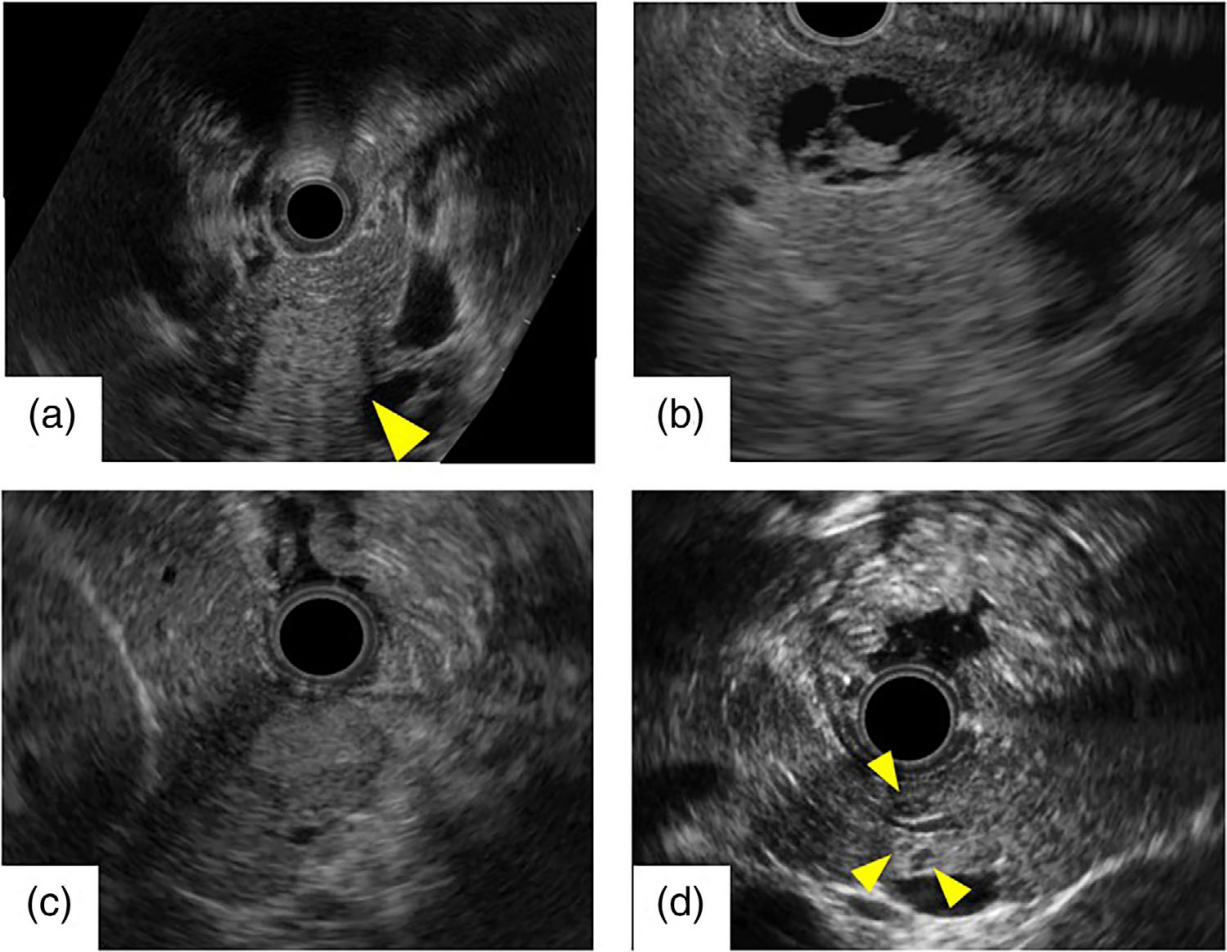
Endoscopic ultrasound. (a) A poorly demarcated hyperechoic lesion that appeared to contain a collection of small cysts was evident in the pancreatic head (yellow arrowhead). (b) A multilocular cystic lesion with thick septa was evident in the pancreatic body. (c) A well‐demarcated, hyperechoic mass was evident in the pancreatic tail. Its interior was homogenous, and it was considered to be a solid mass. (d) Well‐demarcated, isoechoic to hyperechoic lesions a few millimeters in size that had not been identified on contrast‐enhanced computed tomography were present in the pancreas (yellow arrowhead).

**FIGURE 4:**
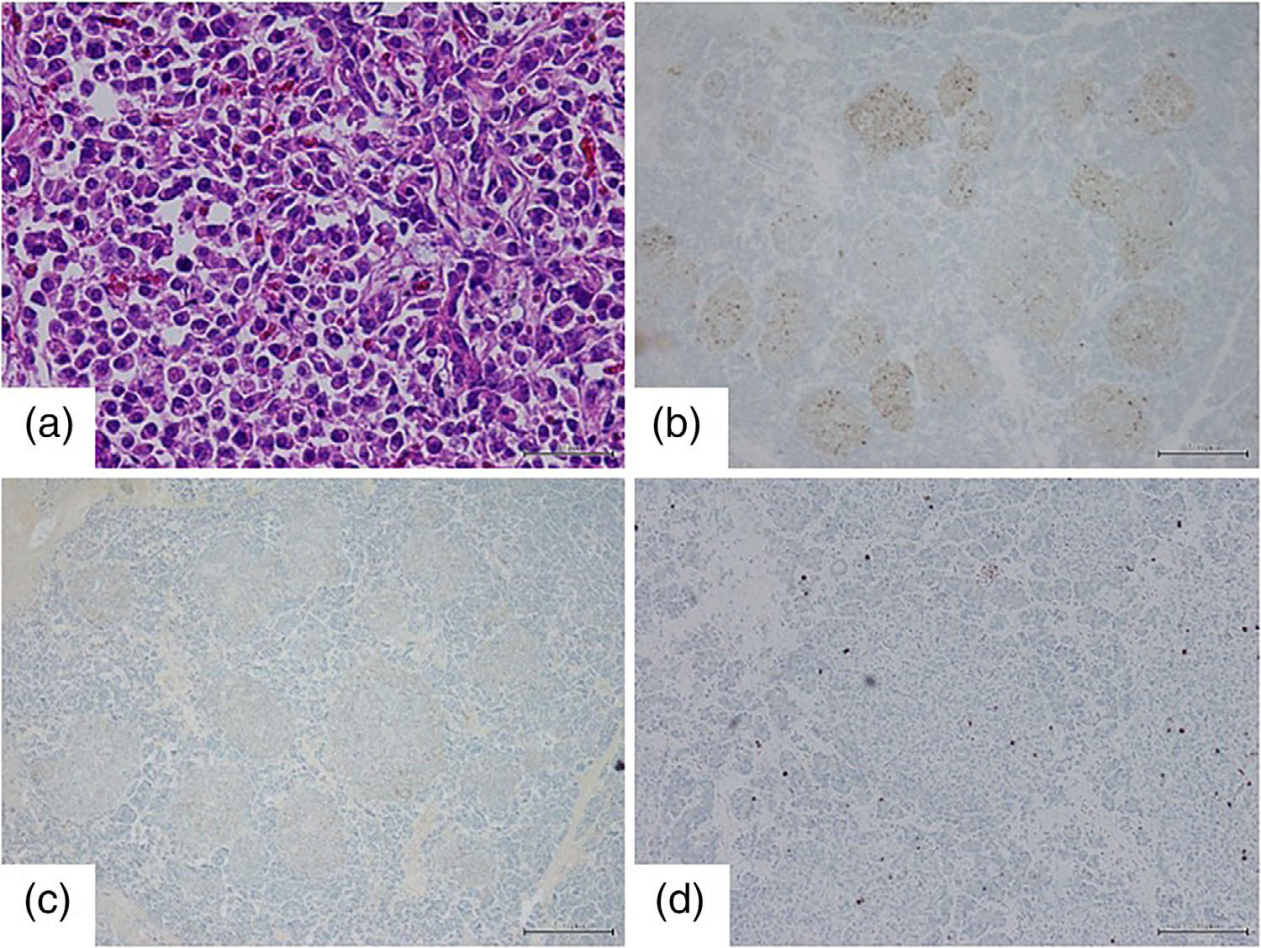
Surgical specimen (pancreatic head lesion). (a) Hematoxylin–eosin staining revealed that the tumor cells were arranged in cords, and had uniformly round nuclei. (b–d) Tumor cells were positive for chromogranin A and glucagon, with a Ki‐67 index of 1%.

On the basis of these imaging findings, multiple neuroendocrine tumors were suspected. Multiple endocrine neoplasia type 1 (MEN1) was ruled out through family history, absence of pituitary adenoma, and absence of hyperparathyroidism. The patient was admitted for an endoscopic ultrasound (EUS)‐guided fine‐needle aspiration. On EUS (Figure 3), the lesion in the pancreatic head was visible as a poorly demarcated hyperechoic area that appeared to contain a collection of small cysts. The lesion in the pancreatic body was a multilocular cystic lesion with 4‐mm‐thick septa. The lesion in the pancreatic tail was a well‐demarcated, hyperechoic mass that exhibited a strong contrast effect from the early phase under perflubutane contrast enhancement. Surprisingly, the pancreas also contained scattered isoechoic to hyperechoic lesions a few millimeters in size that had not been identified on the previously performed contrast‐enhanced CT. We conducted a EUS‐fine‐needle aspiration of the solid nodule in the pancreatic tail. Hematoxylin–eosin staining revealed atypical cells with anisokaryotic, pyknotic, round nuclei, and eosinophilic cell bodies, while immunostaining was positive for chromogranin A with a Ki‐67 index of 1.5%. Therefore, a grade 1 neuroendocrine tumor was diagnosed. Although glucagon staining was negative, glucagonoma was suspected due to the high level of serum glucagon. Somatostatin scintigraphy was performed to screen for distant metastasis; uptake was observed in the lesions in the pancreatic head and tail with no obvious extrapancreatic lesions, suggesting that the lesions were localized within the pancreas. Because of these findings, we decided to conduct a total pancreatectomy and splenectomy for pancreatic multiple neuroendocrine tumors suspected to be glucagonomas. The hematoxylin–eosin staining of the surgical specimen revealed tumor cells in nodules of varying sizes and geographical patterns in every slice. Tumor cells were arranged in cords and had uniformly round nuclei. They were positive for both chromogranin A and glucagon. The Ki‐67 index was 1% (Figure 4). Therefore, a definitive diagnosis of grade 1 pancreatic multiple glucagonoma was made. EUS showed a poorly demarcated lesion in the pancreatic head that appeared to be a cluster of small cysts. Pathology examination revealed several small neuroendocrine tumors, which consist of a cluster of small cysts. The pathology specimen of the cystic lesion from the body of the pancreas was further found to contain cysts, suggesting cystic degeneration with necrosis. The lesion in the pancreatic tail was substantial and a typical neuroendocrine tumor. Currently, 14 months postoperatively, the patient is living recurrence‐free. After the surgery, serum glucagon levels showed a lower value in the detectable range.

## DISCUSSION

Glucagonoma is an extremely rare tumor that accounts for approximately 1%–2% of neuroendocrine tumors.^2^ Currently, 54.7% of glucagonomas are diagnosed when they are in an advanced stage with metastasis.^5^ Wermers et al. described 21 cases of glucagonoma and reported that the most common symptom leading to its discovery was weight loss (71%), followed by necrolytic migratory erythema (67%), diabetes (38%), and diarrhea (29%). Furthermore, patients with glucagonoma have been known to show hyperglucagonemia, anemia, and hypoaminoacidemia.^4^ In our patient, serum glucagon was elevated, and imaging findings demonstrated the presence of multiple neuroendocrine tumors in the pancreas. Even though pathological investigations resulted in a diagnosis of glucagonoma, our patient did not exhibit weight loss or cutaneous lesions, and glucose tolerance was normal. Along with elevated serum glucagon, her blood test results also included hyperaminoacidemia. In our hospital, glucagon levels could only be measured by radioimmunoassay, and as this method only uses glucagon C‐terminal‐recognizing antibodies, it reportedly results in a cross‐reaction with glicentin (1–61) and other glucagon‐related peptides.^6^ Therefore, we requested an external laboratory to conduct glucagon testing with the sandwich enzyme‐linked immunosorbent assay (ELISA) method, which uses both *N*‐terminal‐recognizing and C‐terminal‐recognizing antibodies and has better specificity for glucagon than radioimmunoassay. Thus, hyperglucagonemia was confirmed with the sandwich ELISA method. From these results, we assumed that glucagon cell hyperplasia and neoplasia in this patient might be due to glucagon receptor abnormality. Previous studies reported that biallelic‐inactivating mutations in the glucagon receptor gene cause glucagon cell hyperplasia and neoplasia also termed Mahvash syndrome.^7^, ^8^ In glucagonoma caused by MEN1 and similar conditions, excessive glucagon production promotes glycogenolysis and gluconeogenesis from amino acids, causing abnormal hyperglycemia and hypoaminoacidemia. On the other hand, Mahvash syndrome is known to reduce glycogenolysis and gluconeogenesis from amino acids, resulting in hypoglycemia and hyperaminoacidemia. These suggested consistent association with our patient.

To date, there have been six reported cases of disease caused by biallelic mutations of the glucagon receptor gene,^7^ in which diminished glucagon receptor function causes functional promotion and proliferation of pancreatic α‐cells as a negative feedback loop; it is conjectured that this hyperplasia may progress to neoplasia. The presence of multiple masses in the pancreas in five of the six patients, as in the present patient, may dictate this presumption. Functional analysis of the glucagon receptor showed a loss of function; hence, Mahvash syndrome was diagnosed. In the present case, as multiple pancreatic masses were identified on CT/MRI, we conducted EUS for further investigation. This revealed numerous lesions a few millimeters in size that had not been identified on CT/MRI, and the presence of tumors was also confirmed in the surgical specimen. All lesions identified only by EUS were neuroendocrine tumors sized 2–9 mm, suggesting a high sensitivity of detection by EUS, which is known to be very useful for visualizing small masses.^9^ In a retrospective single‐center cohort study, Khashab et al. reported that EUS could detect 91% of CT‐negative pancreatic neuroendocrine tumors.^10^ From these results, we consider that EUS is the essential test for the detection and in determining surgical technique for glucagonomas.

## CONFLICT OF INTEREST

None.
